# 4-Hy­droxy-6-methyl-3-[3-(thio­phen-2-yl)acrylo­yl]-2*H*-pyran-2-one

**DOI:** 10.1107/S1600536813003826

**Published:** 2013-03-09

**Authors:** Salima Thabti, Amel Djedouani, Abderrahmen Bendaas, Sihem Boufas, Rémi Loui, Dominique Mandon

**Affiliations:** a Laboratoire d’Électrochimie des Matériaux Moléculaires et Complexes, Centre Univeresitaire de B.B.A., Algeria; b Ecole Normale Superieure de Constantine, 25000 Constantine, Algeria; c Université 20 Aout 1955, 21000 Skikda, Algeria; d Laboratoire de Chimie Biomimétique des Métaux de Transition, Institut de Chimie, Strasbourg, France

## Abstract

The title compound, C_13_H_10_O_4_S, crystallizes with two mol­ecules in the asymmetric unit in which the rings make dihedral angles of 3.9 (1) and 6.0 (1)°; this planarity is due in part to the presence of an intra­molecular O—H⋯O hydrogen bond, which generates an *S*(6) ring in each mol­ecule. Both mol­ecules represent *E* isomers with respect to the central C=C bond. In the crystal, mol­ecules are linked by C—H⋯O inter­actions into a three-dimensional network.

## Related literature

For pharmacological properties of chalcones, see: Wattenberg *et al.* (1994 ▶); Dinkova-Kostova *et al.* (1998 ▶); Ram *et al.* (2000 ▶); Kidwai *et al.* (2001 ▶); Ballesteros *et al.* (1995 ▶). For their non-linear optical properties, see: Fichou *et al.* (1988 ▶) and for their importance, see: Tomazela *et al.* (2000 ▶). For precursors in the synthesis of flavonoids, see: Drexler & Amiridis (2003 ▶). For graph-set notation, see: Bernstein *et al.* (1995 ▶). For standard bond lengths, see: Allen *et al.* (1987 ▶).
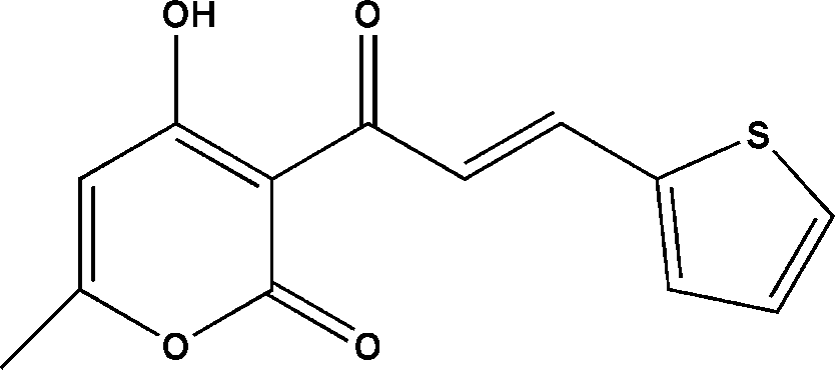


## Experimental

### 

#### Crystal data

C_13_H_10_O_4_S
*M* *_r_* = 262.27
Triclinic, 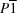

*a* = 8.0737 (4) Å
*b* = 9.9428 (5) Å
*c* = 15.0887 (8) Å
α = 87.770 (1)°
β = 87.779 (3)°
γ = 80.678 (4)°
*V* = 1193.70 (11) Å^3^
*Z* = 4
Mo *K*α radiation
μ = 0.27 mm^−1^
*T* = 293 K
0.5 × 0.4 × 0.2 mm

#### Data collection

Nonius KappaCCD diffractometer
Absorption correction: multi-scan (*SADABS*; Sheldrick, 2002 ▶) *T*_min_ = 0.875, *T*_max_ = 0.947
25106 measured reflections
6954 independent reflections
5295 reflections with *I* > 2σ(*I*)
*R*_int_ = 0.036

#### Refinement

*R*[*F* ^2^ > 2σ(*F* ^2^)] = 0.054
*wR*(*F* ^2^) = 0.157
*S* = 1.06
6954 reflections
326 parameters
H-atom parameters constrained
Δρ_max_ = 0.97 e Å^−3^
Δρ_min_ = −0.30 e Å^−3^

### 

Data collection: *COLLECT* (Nonius, 2002 ▶); cell refinement: *DENZO-SMN* (Otwinowski & Minor, 1997 ▶); data reduction: *EVALCCD* (Duisenberg *et al.*, 2003 ▶); program(s) used to solve structure: *SIR97* (Altomare *et al.*, 1999 ▶); program(s) used to refine structure: *SHELXL97* (Sheldrick, 2008 ▶); molecular graphics: *ORTEP-3 for Windows* (Farrugia, 2012 ▶) and *Mercury* (Macrae *et al.*, 2006 ▶); software used to prepare material for publication: *WinGX* (Farrugia, 2012 ▶) and *PARST* (Nardelli, 1995 ▶).

## Supplementary Material

Crystal structure: contains datablock(s) I, global. DOI: 10.1107/S1600536813003826/ld2095sup1.cif

Structure factors: contains datablock(s) I. DOI: 10.1107/S1600536813003826/ld2095Isup2.hkl

Supplementary material file. DOI: 10.1107/S1600536813003826/ld2095Isup3.cml

Additional supplementary materials:  crystallographic information; 3D view; checkCIF report

## Figures and Tables

**Table 1:** Hydrogen-bond geometry (Å, °)

*D*—H⋯*A*	*D*—H	H⋯*A*	*D*⋯*A*	*D*—H⋯*A*
O3—H33⋯O4	0.82	1.68	2.421 (2)	150
O7—H77⋯O8	0.82	1.65	2.400 (2)	150
C8—H8⋯O5	0.93	2.60	3.481 (3)	159
C10—H10⋯O6	0.93	2.59	3.280 (3)	132
C10—H10⋯O8^i^	0.93	2.59	3.288 (3)	132
C13—H13*C*⋯O6^ii^	0.96	2.58	3.507 (3)	164
C23—H23⋯O4^iii^	0.93	2.56	3.245 (3)	131
C25—H25⋯O7^iv^	0.93	2.38	3.240 (3)	153
C26—H26*B*⋯O1^v^	0.96	2.41	3.338 (3)	163

Symmetry codes: (i) 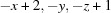; (ii) 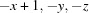; (iii) 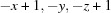; (iv) 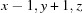; (v) 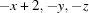.

**Fig. 1.**
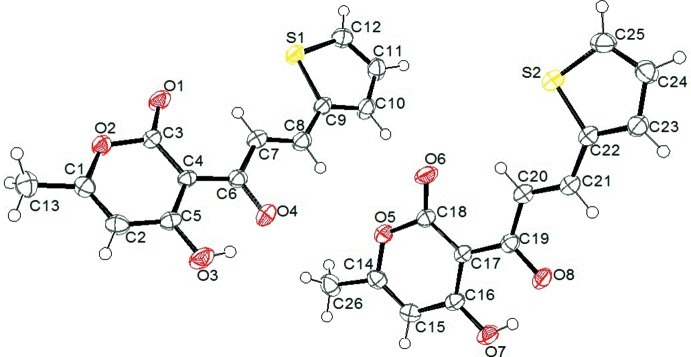
The structure of the title compound in 50% probability ellipsoids.

**Fig. 2.**
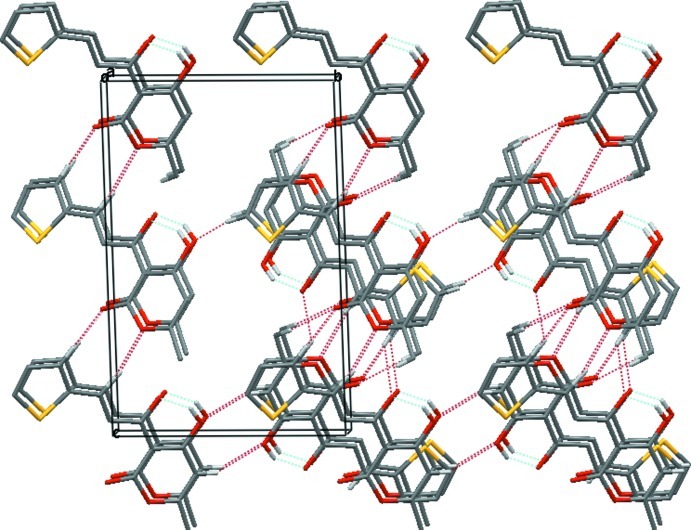
Part of the crystal structure of (I), showing the formation of S(6) rings with dashed bleu lines. C—H···O Hydrogen bonds are shown as red dashed lines. Hydrogen atoms not involved in the motif have been omitted for clarity.

## supplementary crystallographic information

### Comment

Chalcones are an important group of natural products (Tomazela, *et al.*, 2000), which have various pharmacological properties. Some chalcones possess anticancer (Wattenberg, *et al.*, 1994, Dinkova-Kostova, *et al.*, 1998), antimalarial (Ram, *et al.*, 2000), antimicrobial (Kidwai, *et al*., 2001) and anti-inflammatory (Ballesteros, *et al.*, 1995) activities. Chalcones also serve as precursors in the syntheses of different classes of flavonoids (Drexler, *et al.*, 2003). Chalcone derivatives are also a class of organic compounds with excellent NLO properties (Fichou, *et al.*, 1988), much better than those observed in inorganic crystals. Furthermore, several α,β-unsaturated ketones have been found to exhibit biological activity. In this paper, we report a structure containing both thiazole and α,β-unsaturated ketone moieties in one molecule. The crystals do not exhibit second-order nonlinear optical properties as they crystallize in a centrosymmetric space group.

The title compound (Fig. 1) exists in an E configuration with respect to the C7—C8 and C20—C21 double bonds. 6-membered rings adopt a planar conformation. In the cyclic moiety with O2 atom, the r.m.s. deviation for the non-H atoms is 0.0154 Å, with the maximum deviation of C4 from the mean plane being 0.0222 (14) Å. In the moiety with O5 atom, the r.m.s. deviation for the non-H atoms is 0.0063 Å, with the maximum deviation of C17 from the mean plane -0.0105 (13) Å. The thiazole rings are also planar with a r.m.s deviation for non-H atoms of 0.0076 Å (ring with S1) & 0.0020 Å (ring with S2) with a maximum deviation of C9 from the mean plane being (-0.0107 (13)) Å and a maximum deviation of C24 from the mean plane of 0.0029 (15) Å respectively. With respect to the C7—C8 and C20—C21 bonds, the atom pairs C6/C9, H7/H8 and C19/C20, H20/H21 are all *trans*, shown by the value of (-0.05 (14)°) for O8—C21—C20—C19 and 0.42 (15)°) for C6—C7—C8—O4 - 0.42 (15)° torsion angle. The double-bond character of the bond between C7 and C8 is deduced from the short bond distance [1.339 (3) Å]. The value in the other molecule is 1.339 (3) Å for C20/C21]. All bond lengths and angles in (I) have normal values (Allen, *et al.* 1987).

The two rings derived from DHA and 2-Acetyl-thiophene; exhibit coplanar geometry, the coplanarity of the two rings is due to the presence of intramolecular O7—H77···O8 and O3—H33···O4 hydrogen bond. This interactions generate an S(6) ring motif (Bernstein, *et al.* 1995). The overall structure is held by seven C—H···O interactions, forming a three- dimensional network. (Fig. 2). Among others, C25—H25···O7 interaction is the strongest one.

### Experimental

Dehydroacetic acid (0.168 g, 1 mmol) in 1 ml of piperidine was added to thiofene-2-carboxaldehyde (0.112 g, 1 mmol) of in 25 ml of chloroform and then refluxed with stirring under nitrogen atmosphere for 3 days. After that, 5–7 ml of the chloroform-water azeotrope mixture were removed by simple distillation. The product were obtained by slow evaporation of the remaining chloroform and washed with ethyl acetate (2x5ml), then it was recrystalized from dichlromethane and dried under vaccum in dissicator for 24 h (yield 63%). Melting point: 150 °C.

### Refinement

C—H and O—H hydrogen atoms were placed in calculated positions and refined as riding atoms with C—H distances of 0.93 Å with *U*_iso_(H) = 1.2Ueq(C) and O—H distances of 0.82 Å, with *U*_iso_(H) = 1.2Ueq(N).

The methyl H atoms were constrained to an ideal geometry (C—H = 0.96 Å) with *U*_iso_(H) = 1.2Ueq(C), but were allowed to rotate freely about the C—C bonds.

### Crystal data

**Table:**

C_13_H_10_O_4_S	*Z* = 4
*M**_r_* = 262.27	*F*(000) = 544
Triclinic, *P*1	*D*_x_ = 1.459 Mg m^−^^3^
Hall symbol: -P 1	Mo *K*α radiation, λ = 0.71073 Å
*a* = 8.0737 (4) Å	Cell parameters from 102968 reflections
*b* = 9.9428 (5) Å	θ = 2.9–27.5°
*c* = 15.0887 (8) Å	µ = 0.27 mm^−^^1^
α = 87.770 (1)°	*T* = 293 K
β = 87.779 (3)°	Block, brown
γ = 80.678 (4)°	0.5 × 0.4 × 0.2 mm
*V* = 1193.70 (11) Å^3^	

### Data collection

**Table:**

Nonius KappaCCD diffractometer	6954 independent reflections
Radiation source: Enraf Nonius FR590	5295 reflections with *I* > 2σ(*I*)
Graphite monochromator	*R*_int_ = 0.036
Detector resolution: 9 pixels mm^-1^	θ_max_ = 30.0°, θ_min_ = 2.1°
CCD rotation images, thin slices scans	*h* = −11→11
Absorption correction: multi-scan (*SADABS*; Sheldrick, 2002)	*k* = −13→13
*T*_min_ = 0.875, *T*_max_ = 0.947	*l* = −21→21
25106 measured reflections	

### Refinement

**Table:**

Refinement on *F*^2^	Primary atom site location: structure-invariant direct methods
Least-squares matrix: full	Secondary atom site location: difference Fourier map
*R*[*F*^2^ > 2σ(*F*^2^)] = 0.054	Hydrogen site location: inferred from neighbouring sites
*wR*(*F*^2^) = 0.157	H-atom parameters constrained
*S* = 1.06	*w* = 1/[σ^2^(*F*_o_^2^) + (0.0755*P*)^2^ + 0.747*P*] where *P* = (*F*_o_^2^ + 2*F*_c_^2^)/3
6954 reflections	(Δ/σ)_max_ = 0.001
326 parameters	Δρ_max_ = 0.97 e Å^−^^3^
0 restraints	Δρ_min_ = −0.30 e Å^−^^3^

### Special details

**Table:**

Geometry. All e.s.d.'s (except the e.s.d. in the dihedral angle between two l.s. planes) are estimated using the full covariance matrix. The cell e.s.d.'s are taken into account individually in the estimation of e.s.d.'s in distances, angles and torsion angles; correlations between e.s.d.'s in cell parameters are only used when they are defined by crystal symmetry. An approximate (isotropic) treatment of cell e.s.d.'s is used for estimating e.s.d.'s involving l.s. planes.

### Fractional atomic coordinates and isotropic or equivalent isotropic displacement parameters (Å^2^)

**Table:**

	*x*	*y*	*z*	*U*_iso_*/*U*_eq_	
S2	0.46904 (7)	0.30887 (5)	0.54080 (4)	0.03338 (14)	
S1	0.88623 (7)	0.31448 (5)	0.04850 (4)	0.03337 (14)	
O2	0.57005 (19)	−0.13416 (14)	−0.19215 (9)	0.0300 (3)	
O4	0.7556 (2)	−0.19703 (16)	0.10407 (10)	0.0363 (3)	
O3	0.6293 (2)	−0.36848 (16)	0.03371 (11)	0.0417 (4)	
H33	0.6698	−0.3296	0.0725	0.063*	
O5	0.99995 (19)	−0.14198 (15)	0.30123 (10)	0.0311 (3)	
O8	0.8369 (2)	−0.18574 (16)	0.60568 (10)	0.0351 (3)	
O7	1.0375 (2)	−0.35666 (17)	0.53507 (11)	0.0423 (4)	
H77	0.9792	−0.3154	0.5741	0.063*	
O6	0.8273 (2)	0.03013 (16)	0.35590 (11)	0.0415 (4)	
O1	0.6537 (2)	0.04135 (16)	−0.13444 (11)	0.0400 (4)	
C17	0.9154 (2)	−0.15767 (19)	0.45626 (13)	0.0247 (4)	
C4	0.6592 (2)	−0.15850 (18)	−0.04027 (12)	0.0237 (4)	
C1	0.5297 (3)	−0.2617 (2)	−0.18823 (14)	0.0301 (4)	
C5	0.6115 (3)	−0.2898 (2)	−0.03754 (14)	0.0281 (4)	
C2	0.5453 (3)	−0.3400 (2)	−0.11347 (15)	0.0324 (4)	
H2	0.5133	−0.4258	−0.1114	0.039*	
C9	0.9119 (3)	0.1816 (2)	0.12658 (13)	0.0296 (4)	
C3	0.6316 (2)	−0.0752 (2)	−0.12040 (13)	0.0263 (4)	
C22	0.5087 (3)	0.1834 (2)	0.62381 (13)	0.0277 (4)	
C14	1.0980 (3)	−0.2661 (2)	0.30732 (14)	0.0300 (4)	
C25	0.3326 (3)	0.4103 (2)	0.60884 (17)	0.0385 (5)	
H25	0.275	0.4954	0.5916	0.046*	
C19	0.8208 (2)	−0.1100 (2)	0.53485 (13)	0.0269 (4)	
C18	0.9056 (3)	−0.0812 (2)	0.37306 (13)	0.0275 (4)	
C16	1.0214 (3)	−0.2859 (2)	0.46114 (14)	0.0291 (4)	
C10	0.9849 (3)	0.2172 (2)	0.20074 (14)	0.0346 (5)	
H10	1.0118	0.1582	0.2493	0.042*	
C6	0.7344 (2)	−0.1158 (2)	0.03667 (13)	0.0269 (4)	
C21	0.6233 (3)	0.0584 (2)	0.61300 (14)	0.0291 (4)	
H21	0.6401	−0.0005	0.6624	0.035*	
C23	0.4178 (3)	0.2221 (2)	0.69960 (14)	0.0325 (4)	
H23	0.4214	0.1682	0.7515	0.039*	
C20	0.7082 (2)	0.0192 (2)	0.53790 (13)	0.0281 (4)	
H20	0.6947	0.0754	0.4871	0.034*	
C15	1.1117 (3)	−0.3389 (2)	0.38406 (15)	0.0321 (4)	
H15	1.18	−0.4237	0.3869	0.038*	
C7	0.7857 (3)	0.0168 (2)	0.04186 (14)	0.0307 (4)	
H7	0.77	0.0782	−0.0063	0.037*	
C13	0.4714 (3)	−0.2980 (3)	−0.27485 (17)	0.0445 (6)	
H13A	0.4448	−0.3887	−0.2704	0.067*	
H13B	0.5586	−0.2938	−0.3195	0.067*	
H13C	0.3732	−0.2349	−0.2907	0.067*	
C24	0.3181 (3)	0.3516 (2)	0.69120 (16)	0.0374 (5)	
H24	0.25	0.3927	0.7369	0.045*	
C12	0.9682 (3)	0.4177 (2)	0.11694 (16)	0.0383 (5)	
H12	0.9796	0.5076	0.1023	0.046*	
C26	1.1800 (3)	−0.3059 (3)	0.22020 (16)	0.0414 (5)	
H26A	1.2477	−0.3943	0.2264	0.062*	
H26B	1.2496	−0.2403	0.2004	0.062*	
H26C	1.0955	−0.3088	0.1776	0.062*	
C11	1.0149 (3)	0.3533 (2)	0.19538 (15)	0.0377 (5)	
H11	1.0615	0.3944	0.2405	0.045*	
C8	0.8554 (3)	0.0524 (2)	0.11472 (14)	0.0318 (4)	
H8	0.8688	−0.0112	0.1618	0.038*	

### Atomic displacement parameters (Å^2^)

**Table:**

	*U*^11^	*U*^22^	*U*^33^	*U*^12^	*U*^13^	*U*^23^
S2	0.0367 (3)	0.0325 (3)	0.0306 (3)	−0.0057 (2)	−0.0036 (2)	0.0072 (2)
S1	0.0414 (3)	0.0313 (3)	0.0277 (3)	−0.0072 (2)	−0.0059 (2)	0.00652 (19)
O2	0.0375 (8)	0.0285 (7)	0.0251 (7)	−0.0092 (6)	−0.0039 (6)	0.0036 (5)
O4	0.0454 (9)	0.0397 (8)	0.0232 (7)	−0.0067 (7)	−0.0021 (6)	0.0069 (6)
O3	0.0556 (10)	0.0352 (8)	0.0370 (9)	−0.0179 (7)	−0.0078 (7)	0.0151 (7)
O5	0.0372 (8)	0.0296 (7)	0.0260 (7)	−0.0057 (6)	0.0022 (6)	0.0035 (6)
O8	0.0415 (9)	0.0384 (8)	0.0233 (7)	−0.0013 (7)	−0.0040 (6)	0.0069 (6)
O7	0.0514 (10)	0.0375 (8)	0.0315 (8)	0.0106 (7)	−0.0033 (7)	0.0088 (6)
O6	0.0513 (10)	0.0331 (8)	0.0341 (8)	0.0057 (7)	0.0066 (7)	0.0135 (6)
O1	0.0604 (11)	0.0289 (7)	0.0338 (8)	−0.0165 (7)	−0.0139 (7)	0.0116 (6)
C17	0.0250 (9)	0.0250 (8)	0.0243 (9)	−0.0052 (7)	−0.0034 (7)	0.0028 (7)
C4	0.0244 (9)	0.0227 (8)	0.0235 (9)	−0.0036 (6)	0.0014 (7)	0.0035 (7)
C1	0.0271 (9)	0.0295 (9)	0.0345 (11)	−0.0064 (8)	−0.0013 (8)	−0.0029 (8)
C5	0.0283 (9)	0.0257 (9)	0.0301 (10)	−0.0057 (7)	0.0017 (7)	0.0060 (7)
C2	0.0319 (10)	0.0256 (9)	0.0407 (12)	−0.0084 (8)	−0.0013 (8)	0.0013 (8)
C9	0.0350 (10)	0.0291 (9)	0.0247 (9)	−0.0054 (8)	−0.0039 (8)	0.0043 (7)
C3	0.0277 (9)	0.0269 (9)	0.0243 (9)	−0.0048 (7)	−0.0020 (7)	0.0033 (7)
C22	0.0283 (9)	0.0285 (9)	0.0267 (9)	−0.0068 (7)	−0.0028 (7)	0.0037 (7)
C14	0.0300 (10)	0.0272 (9)	0.0349 (11)	−0.0105 (8)	0.0010 (8)	−0.0028 (8)
C25	0.0370 (12)	0.0305 (10)	0.0457 (13)	0.0024 (9)	−0.0084 (10)	0.0001 (9)
C19	0.0272 (9)	0.0281 (9)	0.0264 (9)	−0.0072 (7)	−0.0043 (7)	0.0017 (7)
C18	0.0287 (9)	0.0271 (9)	0.0268 (9)	−0.0059 (7)	0.0006 (7)	0.0035 (7)
C16	0.0298 (10)	0.0286 (9)	0.0287 (10)	−0.0044 (8)	−0.0058 (8)	0.0044 (8)
C10	0.0419 (12)	0.0382 (11)	0.0255 (10)	−0.0130 (9)	−0.0025 (8)	0.0048 (8)
C6	0.0261 (9)	0.0274 (9)	0.0258 (9)	−0.0003 (7)	0.0011 (7)	0.0009 (7)
C21	0.0301 (10)	0.0296 (9)	0.0279 (10)	−0.0064 (8)	−0.0030 (8)	0.0044 (8)
C23	0.0341 (11)	0.0337 (10)	0.0292 (10)	−0.0050 (8)	−0.0010 (8)	0.0020 (8)
C20	0.0271 (9)	0.0293 (9)	0.0278 (10)	−0.0045 (7)	−0.0031 (7)	0.0040 (7)
C15	0.0309 (10)	0.0272 (9)	0.0372 (11)	−0.0019 (8)	−0.0014 (8)	−0.0007 (8)
C7	0.0330 (10)	0.0292 (9)	0.0293 (10)	−0.0046 (8)	−0.0019 (8)	0.0049 (8)
C13	0.0505 (14)	0.0449 (13)	0.0406 (13)	−0.0123 (11)	−0.0105 (11)	−0.0052 (10)
C24	0.0352 (11)	0.0369 (11)	0.0383 (12)	0.0001 (9)	0.0007 (9)	−0.0060 (9)
C12	0.0479 (13)	0.0301 (10)	0.0389 (12)	−0.0129 (9)	−0.0022 (10)	0.0023 (9)
C26	0.0508 (14)	0.0373 (11)	0.0379 (12)	−0.0143 (10)	0.0099 (10)	−0.0064 (9)
C11	0.0468 (13)	0.0400 (11)	0.0293 (11)	−0.0156 (10)	−0.0020 (9)	−0.0037 (9)
C8	0.0346 (11)	0.0321 (10)	0.0274 (10)	−0.0034 (8)	0.0013 (8)	0.0042 (8)

### Geometric parameters (Å, º)

**Table:**

S2—C25	1.708 (2)	C22—C21	1.435 (3)
S2—C22	1.735 (2)	C14—C15	1.340 (3)
S1—C12	1.707 (2)	C14—C26	1.487 (3)
S1—C9	1.728 (2)	C25—C24	1.361 (3)
O2—C1	1.358 (2)	C25—H25	0.93
O2—C3	1.396 (2)	C19—C20	1.450 (3)
O4—C6	1.273 (2)	C16—C15	1.421 (3)
O3—C5	1.303 (2)	C10—C11	1.412 (3)
O3—H33	0.82	C10—H10	0.93
O5—C14	1.356 (3)	C6—C7	1.450 (3)
O5—C18	1.399 (3)	C21—C20	1.339 (3)
O8—C19	1.281 (2)	C21—H21	0.93
O7—C16	1.295 (2)	C23—C24	1.408 (3)
O7—H77	0.82	C23—H23	0.93
O6—C18	1.206 (2)	C20—H20	0.93
O1—C3	1.210 (2)	C15—H15	0.93
C17—C16	1.417 (3)	C7—C8	1.339 (3)
C17—C19	1.439 (3)	C7—H7	0.93
C17—C18	1.440 (3)	C13—H13A	0.96
C4—C5	1.418 (3)	C13—H13B	0.96
C4—C6	1.440 (3)	C13—H13C	0.96
C4—C3	1.443 (3)	C24—H24	0.93
C1—C2	1.344 (3)	C12—C11	1.360 (3)
C1—C13	1.483 (3)	C12—H12	0.93
C5—C2	1.423 (3)	C26—H26A	0.96
C2—H2	0.93	C26—H26B	0.96
C9—C10	1.369 (3)	C26—H26C	0.96
C9—C8	1.450 (3)	C11—H11	0.93
C22—C23	1.368 (3)	C8—H8	0.93
			
C25—S2—C22	91.77 (11)	C9—C10—C11	112.55 (19)
C12—S1—C9	91.51 (10)	C9—C10—H10	123.7
C1—O2—C3	123.26 (16)	C11—C10—H10	123.7
C5—O3—H33	109.5	O4—C6—C4	118.89 (18)
C14—O5—C18	123.20 (16)	O4—C6—C7	117.96 (18)
C16—O7—H77	109.5	C4—C6—C7	123.15 (17)
C16—C17—C19	118.31 (17)	C20—C21—C22	125.64 (19)
C16—C17—C18	118.88 (18)	C20—C21—H21	117.2
C19—C17—C18	122.81 (17)	C22—C21—H21	117.2
C5—C4—C6	118.36 (17)	C22—C23—C24	113.3 (2)
C5—C4—C3	118.30 (17)	C22—C23—H23	123.4
C6—C4—C3	123.34 (17)	C24—C23—H23	123.4
C2—C1—O2	121.62 (19)	C21—C20—C19	120.74 (18)
C2—C1—C13	127.2 (2)	C21—C20—H20	119.6
O2—C1—C13	111.21 (19)	C19—C20—H20	119.6
O3—C5—C4	121.18 (18)	C14—C15—C16	119.67 (19)
O3—C5—C2	118.20 (18)	C14—C15—H15	120.2
C4—C5—C2	120.61 (18)	C16—C15—H15	120.2
C1—C2—C5	119.11 (18)	C8—C7—C6	121.25 (19)
C1—C2—H2	120.4	C8—C7—H7	119.4
C5—C2—H2	120.4	C6—C7—H7	119.4
C10—C9—C8	125.57 (19)	C1—C13—H13A	109.5
C10—C9—S1	111.12 (16)	C1—C13—H13B	109.5
C8—C9—S1	123.26 (15)	H13A—C13—H13B	109.5
O1—C3—O2	114.36 (17)	C1—C13—H13C	109.5
O1—C3—C4	128.67 (18)	H13A—C13—H13C	109.5
O2—C3—C4	116.96 (16)	H13B—C13—H13C	109.5
C23—C22—C21	125.88 (19)	C25—C24—C23	112.6 (2)
C23—C22—S2	110.40 (15)	C25—C24—H24	123.7
C21—C22—S2	123.71 (16)	C23—C24—H24	123.7
C15—C14—O5	121.52 (19)	C11—C12—S1	112.14 (17)
C15—C14—C26	127.2 (2)	C11—C12—H12	123.9
O5—C14—C26	111.28 (19)	S1—C12—H12	123.9
C24—C25—S2	111.97 (17)	C14—C26—H26A	109.5
C24—C25—H25	124	C14—C26—H26B	109.5
S2—C25—H25	124	H26A—C26—H26B	109.5
O8—C19—C17	118.31 (18)	C14—C26—H26C	109.5
O8—C19—C20	118.39 (19)	H26A—C26—H26C	109.5
C17—C19—C20	123.31 (17)	H26B—C26—H26C	109.5
O6—C18—O5	114.24 (18)	C12—C11—C10	112.6 (2)
O6—C18—C17	129.1 (2)	C12—C11—H11	123.7
O5—C18—C17	116.70 (17)	C10—C11—H11	123.7
O7—C16—C17	121.05 (19)	C7—C8—C9	125.68 (19)
O7—C16—C15	118.95 (19)	C7—C8—H8	117.2
C17—C16—C15	120.00 (18)	C9—C8—H8	117.2

### Hydrogen-bond geometry (Å, º)

**Table:**

*D*—H···*A*	*D*—H	H···*A*	*D*···*A*	*D*—H···*A*
O3—H33···O4	0.82	1.68	2.421 (2)	150
O7—H77···O8	0.82	1.65	2.400 (2)	150
C8—H8···O5	0.93	2.60	3.481 (3)	159
C10—H10···O6	0.93	2.59	3.280 (3)	132
C10—H10···O8^i^	0.93	2.59	3.288 (3)	132
C13—H13*C*···O6^ii^	0.96	2.58	3.507 (3)	164
C23—H23···O4^iii^	0.93	2.56	3.245 (3)	131
C25—H25···O7^iv^	0.93	2.38	3.240 (3)	153
C26—H26*B*···O1^v^	0.96	2.41	3.338 (3)	163

Symmetry codes: (i) −*x*+2, −*y*, −*z*+1; (ii) −*x*+1, −*y*, −*z*; (iii) −*x*+1, −*y*, −*z*+1; (iv) *x*−1, *y*+1, *z*; (v) −*x*+2, −*y*, −*z*.
